# Performance Evaluation and Structure Optimization of Low-Emission Mixed Epoxy Asphalt Pavement

**DOI:** 10.3390/ma15186472

**Published:** 2022-09-18

**Authors:** Yulou Fan, You Wu, Huimin Chen, Shinan Liu, Wei Huang, Houzhi Wang, Jun Yang

**Affiliations:** School of Transportation, Southeast University, Nanjing 211189, China

**Keywords:** epoxy asphalt concrete (EAC), low-emission mixed asphalt, pavement structure, finite element method (FEM), mechanical response, life cycle assessment (LCA)

## Abstract

Epoxy asphalt concrete (EAC) has excellent properties such as high strength, outstanding thermal stability, and great fatigue resistance, and is considered to be a long-life pavement material. Meanwhile, the low initial viscosity of the epoxy components provides the possibility to reduce the mixing temperature of SBS-modified asphalt. The purpose of this study is to verify the feasibility of low-emission mixing of SBS-modified epoxy asphalt and to compare the mechanical responses in several typical structures with EAC, in order to perform structure optimization for practical applications of EAC. In this paper, the Brookfield rotational viscosity test was conducted to investigate the feasibility of mixing SBS-modified epoxy asphalt at a reduced temperature. Subsequently, the dynamic modulus tests were carried out on EAC to obtain the Prony series in order to provide viscoelastic parameters for the finite element model. Six feasible pavement structures with EAC were proposed, and a finite element method (FEM) model was developed to analyze and compare the mechanical responses with the conventional pavement structure. Additionally, the design life was predicted and compared to comprehensively evaluate the performance of EAC structures. Finally, life cycle assessment (LCA) on carbon emissions was developed to explore the emission reduction effect of the epoxy asphalt pavement. The results indicate that the addition of epoxy components could reduce the mixing temperature of SBS-modified asphalt by 30 °C. The proper use of EAC can significantly improve the mechanical condition of the pavement and improve its performance and service life. It is recommended to choose S5 (with EAC applied in the middle-lower layer) as the optimal pavement structure, whose allowable load repetitions to limit fatigue cracking were more than 1.7 times that of conventional pavements and it has favorable rutting resistance as well. The LCA results show that in a 25-year life cycle, the carbon emissions of epoxy asphalt pavements could be reduced by 29.8% in comparison to conventional pavements.

## 1. Introduction

With the increasing traffic volume, heavier traffic loads, and higher requirements for pavement, the conventional ordinary asphalt pavement has gradually failed to meet the demands of roads. Numerous pavement distresses, such as rutting, fatigue failure, and thermal cracking, frequently occur before the expected service life; thus, there is a demand for new durable pavement materials. Epoxy asphalt (EA), known as a thermosetting polymer-modified asphalt, consists of thermosetting epoxy resin, curing agent, asphalt binder, and other additives [1]. After mixing and curing, the epoxy resin and the curing agent undergo a cross-linking reaction, forming irreversible cross-linked networks, which greatly restrict the mobility of asphalt molecules, giving the epoxy asphalt thermosetting characteristics [2,3,4]. The cured epoxy asphalt concrete has changed the thermoplastic nature of conventional asphalt and has outstanding physical and mechanical properties such as high strength and stiffness, excellent thermal stability, good corrosion resistance, and superior fatigue resistance [5,6]. Consequently, EAC has been widely used as paving materials for airfields and orthotropic steel bridge decks around the world [7,8,9]. In China, epoxy asphalt was used for the first time on the Nanjing Second Yangtze River Bridge in 2000, where a 50 mm thick dense-graded EAC was paved on the deck [10,11]. In the over 20 years of service so far, the EAC on the Nanjing Second Yangtze River Bridge has shown an amazingly outstanding performance, which has led to the widespread application of EAC on orthotropic steel bridge decks built in China, including the Nansha Bridge (main span 1688 m), the Sutong Yangtze River Bridge (main span 1088 m), and the Xihoumen Bridge (main span 1650 m) [12,13].

Meanwhile, due to the outstanding properties and satisfactory performance in bridge deck paving, researchers have started to experiment with EAC in roadway pavement applications, in which EAC is considered to possess the potential to be a long-life pavement material [14,15,16]. In most performance indicators, epoxy asphalt outperforms ordinary asphalt by at least two times and even more than 10 times in some cases. Lu and Bors [13] reviewed the potential application of epoxy asphalt in roadway engineering, and pointed out that EAC has good fatigue resistance, high-temperature stability, and strong resistance to aging and chemical attack. Xie et al. [17] summarized the factors affecting the mechanical properties, bonding properties, and thermal stability of epoxy asphalt and analyzed the mechanism of phase separation and its effect on the mechanical properties of epoxy asphalt. Chen et al. [16] investigated the physical, chemical, and mechanical properties of EAC and found that it exhibited remarkably higher compressive strength, rutting resistance, durability, and water resistance; however, its cracking resistance at low temperatures was slightly reduced. In other studies, rheological characteristics were studied as an important indicator affecting deformation and construction, and it was found that epoxy asphalt has better rheological properties than conventional modified asphalt, with favorable fluidity and low viscosity before curing, which can effectively fill the voids in the mixture and make it more uniform [7,14,18]. Additionally, the fatigue properties of EAC reflect the durability of the road and is directly related to the service life of asphalt pavements [19]. Due to the high stiffness and bonding properties of epoxy asphalt, EAC has superior fatigue resistance when compared to conventional asphalt concrete, especially at high epoxy resin dosages [20,21]. Li et al. [22] explored the fatigue performance of porous epoxy asphalt mixtures and found that epoxy asphalt binders can significantly improve the resistance to fatigue damage of porous asphalt mixtures but it is relatively sensitive to strain levels. Furthermore, the addition of glass or mineral fibers can further strengthen the fatigue resistance of EAC [23].

Additionally, because the initial viscosity of epoxy components is relatively low compared to asphalt, the viscosity of the epoxy asphalt will gradually increase from a lower value during the curing reaction [14]. This property provides the epoxy asphalt with better workability, especially for SBS-modified asphalt, which allows EAC to be mixed at a lower temperature, thus reducing emissions in the production process. Moreover, due to its excellent performance, EAC generally has a much longer life than the conventional asphalt mixtures and can meet the increasing requirements of low-emission, “green”, and long-life pavements [13]. Furthermore, the most effective measure for emission reduction is to improve the service life of the pavement. From this perspective, epoxy asphalt pavement may also have a more favorable emission reduction effect. In summary, the feasibility of epoxy asphalt for roadway pavement has been demonstrated by numerous researchers based on extensive research and test results.

However, besides the superb properties of the epoxy asphalt itself, pavement structure is also crucial for the application and field performance of new pavement materials [24]. Recently, the finite element method (FEM) has become a popular numerical simulation method for calculating the mechanical response of numerous pavement structures, which can adapt to complex geometries and boundary conditions and is applicable to solve a variety of linear and nonlinear problems [25,26]. Guo et al. [27] explored the dynamic response of five typical pavement structures based on ABAQUS and found that the stresses and strains in the full-depth pavements were less than those in semi-rigid base pavements, but the rutting distress was the opposite. Yu-Shan and Shakiba [28] explored the performance of asphalt pavement under different asphalt concrete and base thicknesses, base and subgrade permeability, and various saturations by using a three-dimensional FEM model and found that the pavement structure has a significant effect on the peeling phenomenon of the asphalt layer. In other studies, researchers have investigated various pavement performance qualities under different structures using FEM, such as mechanical responses, rutting, top-down cracking, and reflective cracking [29,30,31,32]. In summary, the structure combination will greatly influence the mechanical responses and the field performance of the pavement. Thus, the pavement performance of EAC should be evaluated in order to determine the optimal structure before it is widely used in the field. Nevertheless, there are research gaps for the performance evaluation and structure optimization of EAC, which limits its application in engineering practice.

The objective of this study is to verify the low-emission mixed characteristics of epoxy asphalt through viscosity tests, compare the performance of EAC in several typical structures, and select the optimal pavement structure considering the mechanical responses and the design life, in order to provide a reference for the practical application of EAC. Meanwhile, the emission reduction effect of the epoxy asphalt pavement is explored using life cycle assessment (LCA).

## 2. Materials and Methods

### 2.1. Experimental Design

#### 2.1.1. Materials

Styrene–butadiene–styrene (SBS)-modified epoxy asphalt was used in this study. The matrix asphalt was a 60/80 asphalt with the performance grade of PG 64–22, and the SBS was SBS (I-D) produced in China, with a dosage of 5.0%. The epoxy resin and curing agent were produced in China, and the ratio of epoxy resin to curing agent is 1:1. The basic properties and technical indicators of asphalt binder, epoxy resin, and curing agent were tested according to the Chinese specification “Standard Test Methods of Bitumen and Bituminous Mixtures for Highway Engineering” (JTG E20-2011) [33] and shown in Table 1. The ratio of the mass of the epoxy resin components (including the epoxy resin and curing agent) to that of the asphalt was chosen as 3:7, that is, the dosage of epoxy resin components was 30% of the entire epoxy asphalt binder by weight. The gradation of EAC-13, i.e., epoxy asphalt concrete with a nominal maximum aggregate size (NMAS) of 13 mm, is the same as the typical gradation of AC-13 (asphalt concrete with an NMAS of 13 mm), as shown in Table 2. According to the “Technical Specifications for Construction of Highway Asphalt Pavements” (JTG F40-2004) [34], the optimal binder–aggregate ratio for EAC-13 was determined to be 6.3% based on the Marshall method. Two EAC-13 specimens with a height of 170 mm and a diameter of 150 mm were prepared on a shear gyratory compactor, which were cored and cut for dynamic modulus testing with a height of 150 mm and a diameter of 100 mm.

#### 2.1.2. Viscosity Tests

The viscosity–temperature relationship of the asphalt binder is the key to control the workability of the asphalt mixture. The initial viscosity of the epoxy asphalt is reduced after mixing due to the lower viscosity of the epoxy components compared to the asphalt binder. However, it will gradually increase as the curing reaction proceeds. Therefore, the viscosity of epoxy asphalt as a function of time and temperature needs to be investigated to determine its rheological properties and mixing temperatures.

The viscosity of SBS-modified epoxy asphalt was tested at different temperatures (120, 130, 140, and 150 °C) using a Brookfield rotary viscometer (Model DV2T, Brookfield Engineering Inc., Middleboro, MA, USA). The test time was 120 min at a speed of 100 rpm, which was changed to 50 rpm for 120 °C and 130 °C after 80 min in order to keep the rotational torque within the normal range.

#### 2.1.3. Dynamic Modulus Tests

**Figure 1:**
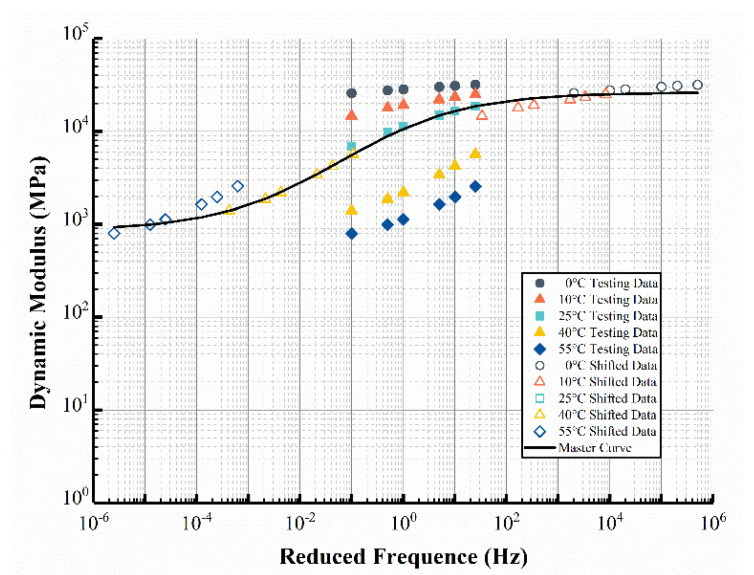
The dynamic modulus master curve of EAC-13.

As a typical viscoelastic material, the mechanical properties of asphalt mixtures exhibit dependency on time and temperature. In order to evaluate the viscoelastic properties of EAC, dynamic modulus tests were carried out by the universal testing machine (UTM-25, IPC Global, CONTROLS, Milan, Italy) using the semi-sine wave loading form, according to the “Standard Test Methods of Bitumen and Bituminous Mixtures for Highway Engineering” (JTG E20-2011) [33]. The dynamic moduli were calculated under 6 test frequencies (0.1, 0.5, 1, 5, 10, and 25 Hz) and 5 test temperatures (0, 10, 25, 40, and 55 °C). The dynamic modulus master curve at reference temperature of 25 °C was constructed using the Sigmoid model (Figure 1), and the time–temperature shift factor $\alpha_{T}\;$was calculated using the Williams–Landel–Ferry (WLF) equation.

The viscoelastic properties of asphalt mixtures in ABAQUS can be characterized by the Prony series. The fitted Prony series parameters of EAC at 25 °C with 11 relaxation components were used for modeling, as shown in Table 3. Due to the strong bonding of epoxy asphalt, gradation has a relatively reduced effect on the properties of EAC, so the same Prony series was applied for EAC-13 and EAC-16. The Prony series of the other asphalt mixtures used in the simulation were determined according to reference [31].

### 2.2. Finite Element Modeling of Different Structures

#### 2.2.1. Pavement Structures

Based on the previous experience, 6 feasible pavement structures with EAC (S1–S6) were proposed in this paper, and 1 conventional structure was selected as the control group (S0). The pavement structure is composed of an asphalt surface layer with a total thickness of 26 cm, a cement-treated base (CTB, 38 cm), a lime-fly ash stabilized soil sub-base (LFS, 20 cm), and soil (SG, 8 m). The pavement width was selected as 42 m, based on a two-way eight-lane highway with a designated speed of 120 km/h (each lane is 3.75 m in width with a 4.5 m central reservation and 3.75 m shoulders on both sides). All pavement structures are summarized in Table 4.

#### 2.2.2. Simulation Method

The two-dimensional ABAQUS models of 7 pavement structures with a size of 42 m × 8.84 m were established, and the material parameters are shown in Table 5. Additionally, the viscoelastic properties were taken into account for the asphalt layers according to the Prony series fitted in Section 2.1.2. The standard axle load BZZ-100 specified in Chinese “Specifications for Design of Highway Asphalt Pavement” (JTG D50-2017) [35] was used to convert the loading area. The double-circle vertical uniform load was set at the top of the pavement, with the equivalent circle radius obtained by Equation (1), and the vertical compressive stress was 0.7 MPa. The distance between the two equivalent circles is 0.1065 m; thus, the width of the loading area, d, is 0.213 m, with an interval of 0.1065 m. The traffic loads were applied at the center of the 8 lanes.

(1) $$\begin{array}{c} d=\sqrt{\frac{4P}{\pi p}}=\sqrt{\frac{4\times \frac{100}{4}}{\pi \times 700000}}=0.213\;\left(m\right) \end{array}$$

where P = axle load on each wheel and p = tire internal pressure, i.e., the contact pressure.

In this study, the pavement structure was simplified to a two-dimensional plane strain problem, so the standard load of 0.7 MPa should be converted into a linear load of 117,371 Pa based on the equivalent static principle. To simulate the dynamic response of the pavement, a half-sine dynamic cyclic load with a period of 0.2 s was applied, according to Equation (2):

(2) $$\begin{array}{c} y=0.11737sin10\pi t\;\left(\mathrm{MPa}\right) \end{array}$$

**Figure 2:**
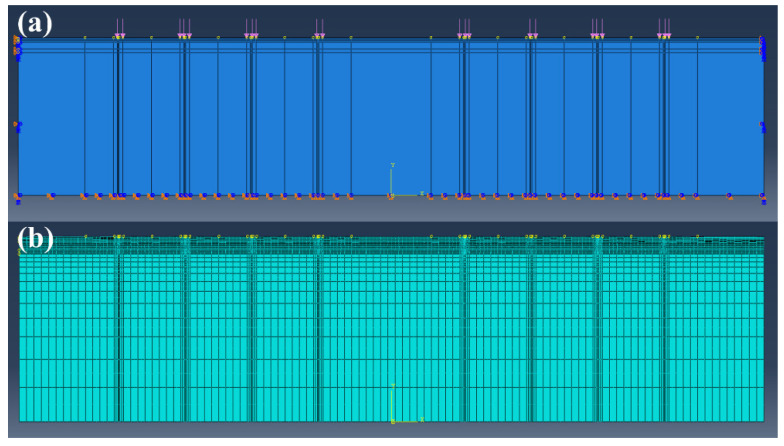
Pavement modeling in ABAQUS: (**a**) loads and boundary conditions; (**b**) meshes.

The boundary conditions are: completely fixed at the bottom edge of the soil and no lateral displacement on both sides of the whole structure. The mesh of the loading area was refined, and the element type is CPE4R, i.e., 4-node continuum bilinear plane strain with reduced integration (Figure 2). The mechanical responses of 7 structures were captured, including the tensile strain at the bottom of the asphalt layer, the tensile stress at the bottom of the base, the vertical compressive strain on the top of the subgrade, and the shear strain of the asphalt layer.

### 2.3. Calculation of Design Life

In order to compare the design life of different pavement structures, load repetitions were calculated using the functions applicable to the response of various types of structures proposed by JTG D50-2017 [35]. The fatigue life of the asphalt layer $N_{f}$ was obtained based on the tensile strain at the bottom of the asphalt layer according to Equation (3):

(3) $$\begin{array}{c} N_{f}=6.32\times 10^{15.96-0.29\beta }k_{a}k_{b}k_{T}^{-1}{\left(\frac{1}{\varepsilon_{a}}\right)}^{3.97}{\left(\frac{1}{E_{a}}\right)}^{1.58}{\left(VFA\right)}^{2.72} \end{array}$$

where $N_{f}$ is the fatigue cracking life of asphalt layer (times), β is the target reliability index (1.65 for freeway), $k_{a}$ is the coefficient for seasonal permafrost areas, and $k_{b}$ is the coefficient of the fatigue loading mode, calculated based on Equation (4):

(4) $$\begin{array}{c} k_{b}={\left[\frac{1+0.03E_{a}{}^{0.43}{\left(VFA\right)}^{-0.85}e^{0.024h_{a}-5.41}}{1+e^{0.024h_{a}-5.41}}\right]}^{3.33} \end{array}$$

where $E_{a}$ is the dynamic modulus of asphalt mixture at 20 °C (MPa), VFA is the voids filled with asphalt (%), $h_{a}$ is the thickness of the asphalt layer, $k_{T}$ is the temperature adjustment coefficient, and $\varepsilon_{a}$ is the tensile strain at the bottom of asphalt layer ($10^{-6}$).

Additionally, in this study, to limit the effects of rutting and subgrade deformation, the load repetitions $N_{d}$ were calculated based on the allowable vertical compressive strain on the top of the subgrade according to Equation (5):

(5) $$\begin{array}{c} \left[\varepsilon_{z}\right]=1.25\times 10^{4-0.1\beta }{\left(k_{T}N_{d}\right)}^{-0.21} \end{array}$$

where $\left[\varepsilon_{z}\right]$ is the allowable vertical compressive strain on the top of the subgrade ($10^{-6}$). Based on the principle that the calculated strain should be less than the allowable strain ($\varepsilon_{z}<\left[\varepsilon_{z}\right]$), $N_{d}$ can be determined.

In this paper, the material parameters $E_{a}$ and VFA were determined by laboratory tests (Section 2.1.3), while the mechanical parameters $\varepsilon_{a}$ and $\varepsilon_{z}$ were obtained from simulation results in Section 3.2. The load repetitions $N_{f}$ and $N_{d}$ calculated above were used to compare the service life of epoxy asphalt pavement with that of conventional asphalt pavement to verify its long-life characteristics.

### 2.4. Life Cycle Assessment (LCA)

To further investigate the carbon reduction effect of epoxy asphalt pavement, a life cycle assessment (LCA) on the carbon emissions of epoxy asphalt pavement and conventional pavement was conducted based on the calculated fatigue life. The service life of conventional asphalt pavement is usually 15 years, while that of epoxy asphalt pavement is 1.7 times longer, according to the results in Section 3.3, up to 25 years or more, so a calculation period of 25 years was used for LCA.

The LCA model on carbon emissions of asphalt pavements can be divided into two main parts, the construction period and the operation period. The construction period includes material production, mixing, transportation, paving, and rolling, while the operation period consists of milling, maintenance and repair, and traffic delays. The basic energy consumption and emissions were referenced from the Chinese Life Cycle Database (CLCD) [36], where the calorific value of the energy consumption process referred to “China Energy Statistical Yearbook 2020” [37], and the carbon emission factors referred to “IPCC Guidelines for National Greenhouse Gas Inventories” [38]. The carbon emissions of material production were available through the references, and the carbon emission factors of equipment used in other processes were obtained by converting from the total power.

In this paper, it is assumed that in the 25-year life cycle for conventional asphalt pavement, a total of 3 major repairs are needed with a milling thickness of 10 cm, as well as 4 minor repairs by adding an ultra-thin overlay with a thickness of 1 cm. For epoxy asphalt pavement, because of its excellent performance and longer fatigue life, only 1 major repair and 2 minor repairs are required. Taking a 1000 m*0.1 m*30 m pavement section as an example, the carbon emissions in a life cycle of epoxy asphalt pavements and conventional asphalt pavements in the construction period and operation period were calculated and compared.

## 3. Results and Discussions

### 3.1. Viscosity Tests

**Figure 3:**
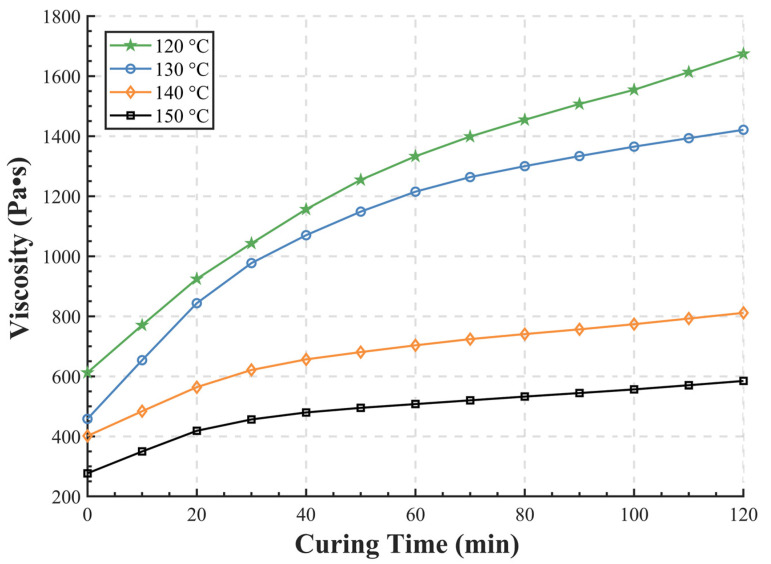
Viscosity test results of SBS-modified epoxy asphalt.

The viscosity–time curves of SBS-modified epoxy asphalt (with a 30% dosage of epoxy components) at different temperatures are shown in Figure 3. It illustrates that the viscosity of epoxy asphalt has a typical dependence on time and temperature. As time increases and the curing reaction develops, the epoxy asphalt gradually acquires thermosetting properties and therefore, the viscosity increases. The curves of viscosity and curing time are consistent with the trends observed in the studies of Cong [39] and Luo [40], where they pointed that the viscosity of hot-mixed epoxy asphalt showed a logarithmic relationship with time. Meanwhile, the initial viscosity of the epoxy asphalt binder reduces with an increase in temperature, and it is 615, 450, 400, and 265 mPa•s at 120, 130, 140, and 150 °C, respectively. This characteristic indicates the importance of temperature in the mixing and construction process.

Additionally, the low initial viscosity of the epoxy component opens up the possibility of lower mixing and compaction temperatures. According to Zhang et al. [41], the suitable viscosities for mixing and compaction of SBS-modified asphalt are (0.32 ± 0.03) Pa•s and (0.45 ± 0.05) Pa•s, respectively. To achieve this viscosity, it is usually necessary to mix the SBS-modified asphalt at around 180 °C, which might result in numerous emissions and the deterioration of the asphalt binder. Nevertheless, from the viscosity test results in Figure 3, the addition of epoxy components provides the SBS-modified epoxy asphalt with a lower viscosity, and the requirements described above can be achieved at 150 °C (0–40 min), thus allowing the mixing process to proceed at a lower temperature. It can also be noted that a mixing temperature below 150 °C might be undesirable, because the viscosity has exceeded 1.0 Pa•s after 30 min of curing at 120 °C or 130 °C, which will cause difficulties in construction and compaction. Additives such as warm mix agents are needed if there is a demand for much lower mixing temperatures.

In summary, it is pleasing to find that the addition of the epoxy components allows the SBS-modified asphalt to be mixed at a lower temperature. The mixing temperature of SBS-modified epoxy asphalt is reduced by 30 °C (from 180 °C to 150 °C), thus achieving a low-emission mixed-asphalt mixture, which has a better temperature reduction effect and meets the low-carbon and emission reduction requirements.

### 3.2. Mechanical Responses of Different Structures

#### 3.2.1. Tensile Strain at the Bottom of Asphalt Layer

**Figure 4:**
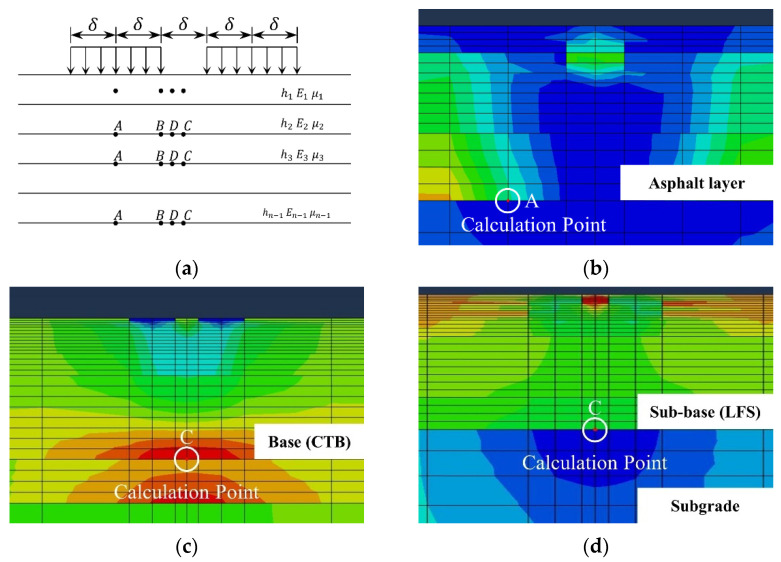
Calculation basis: (**a**) elastic layer system; (**b**) calculation point of tensile strain; (**c**) calculation point of tensile stress; (**d**) calculation point of vertical compressive strain.

**Figure 5:**
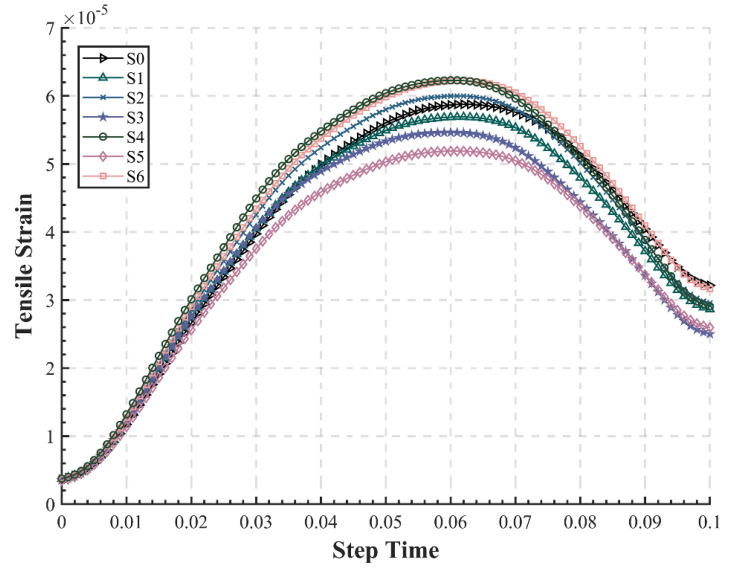
Time–history curve of tensile strain at the bottom of asphalt layer.

The tensile strain at the bottom of the asphalt layer is closely correlated with the fatigue life of the pavement. According to JTG D50-2017 [35], the mechanical response of the asphalt pavement structure can be calculated in the elastic layer system (Figure 4a), so the point A in the inner lane was selected as the calculation point based on the maximum principle (Figure 4b). In this model, point A corresponds to Node 2650, Element 5170 for structures S0, S2, S4, and S6. For structures S1, S3, and S5, point A is Node 2028 and Element 3674. The tensile strains at the bottom of the asphalt layers of seven pavement structures under dynamic loading were extracted and initially filtered, as shown in Figure 5. It demonstrates that under dynamic loading, the mechanical response of the asphalt pavement also varies dynamically; however, it exhibits a hysteresis to some extent. According to Equation (2), the traffic load achieved its maximum value at 0.05 s, but the tensile strain reached the maximum value at approximately 0.06 s. This is because the asphalt mixture was given viscoelastic properties in the simulation, so the external loads would be transferred gradually in the depth direction of the pavement, reflecting the time-delayed effect, which is consistent with the actual situation.

It can be discovered from Figure 5 that structure S5 has the lowest maximum tensile strain at the bottom of asphalt layer of $5{.21\;\times \;10}^{-5}$, while S4 and S6 are similar and highest, exceeding $6{.25\;\times \;10}^{-5}$. The maximum tensile strain and its occurrence time of the seven structures are listed in Table 6. Three conditions (S1, S3, and S5) have significantly lower tensile strains than the conventional pavement S0 without EAC, decreasing by 3.13%, 6.96%, and 11.81%, respectively, showing a better mechanical response, which indicates that structures with EAC might present a longer fatigue life. The structural mechanics study by Chen et al. also obtained fatigue life by controlling the maximum tensile strain at the bottom of the asphalt layer [42]. The values of epoxy asphalt pavements in this paper are much smaller than the simulation results of conventional asphalt pavements of Jiang et al. (around ${9\;\times \;10}^{-5}$ to ${12\;\times \;10}^{-5}$) [43]. Therefore, the placement of EAC can prevent the structure from generating excessive flexural tensile strains, thus delaying the fatigue-induced structural failure of the pavement. Furthermore, the maximum value appears earliest in S0, and the other structures with EAC exhibit a longer time delay, which might suggest that the new structures with EAC have better viscoelasticity and ductility. Meanwhile, the results also indicate that setting a 1 cm AR-SAMI as a stress-absorbing layer can significantly reduce the maximum tensile strain at the bottom of the asphalt layer, which in turn can optimize the stress state and improve the fatigue life of the pavement to a certain extent.

#### 3.2.2. Tensile Stress at the Bottom of Base

**Figure 6:**
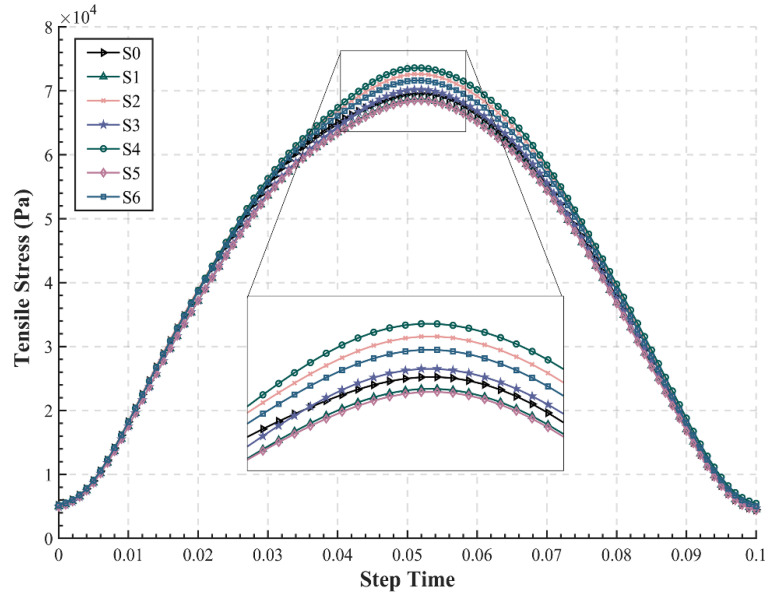
Time–history curve of tensile stress at the bottom of the base (CTB).

As the main bearing layer of the semi-rigid base pavement, the mechanical characteristics of the base are critical to the service life of asphalt pavements. According to the principle of maximum value, the center point in the outermost lane has the maximum tensile stress, so the point C at the bottom of CTB in the outermost lane was chosen as the calculation point of tensile stress (Figure 4c), which was Node 74, Element 1448 for S0, S2, S4, and S6 and Node 423, Element 5371 for S1, S3, and S5. The tensile stress values at the bottom of the base of seven structures were extracted and filtered, as shown in Figure 6. Since the traffic load is a half-sine load, the tensile stress at the bottom of the base also demonstrates a sinusoidal trend with time. It is interesting that the time-delayed effect of tensile stress is much less compared to the tensile strain, which is in line with the viscoelastic characteristics of asphalt pavements, i.e., the deformation will exhibit a significant time delay under continuous loading rather than stress. The maximum tensile stress and its occurrence time in the seven structures are also listed in Table 6.

The maximum tensile stress at the bottom of CTB ranges from 59.7% to 64.2% of the traffic load, which indicates that there is an overall attenuation in the downward transfer of the surface loads (which might first increase before decreasing in the asphalt layer), consistent with accepted perceptions. The analysis in Figure 6 illustrates that S5 still has the minimum tensile stress at the bottom of base with a maximum value of 70,094 Pa, which is similar to S1, and both of them reduce by 1.54% and 1.21% compared to the conventional structure S0. However, the structure S4 shows the largest tensile strain of about 75,352 Pa. This might be due to the fact that the high modulus of EAC applied to the upper layer causes a change in the stress distribution of the pavement, which makes the stress more dispersed in the lateral direction and influenced by the adjacent lanes, thus increasing the tensile stress of the base.

#### 3.2.3. Maximum Strain and Stress along Depth of the Pavement

**Figure 7:**
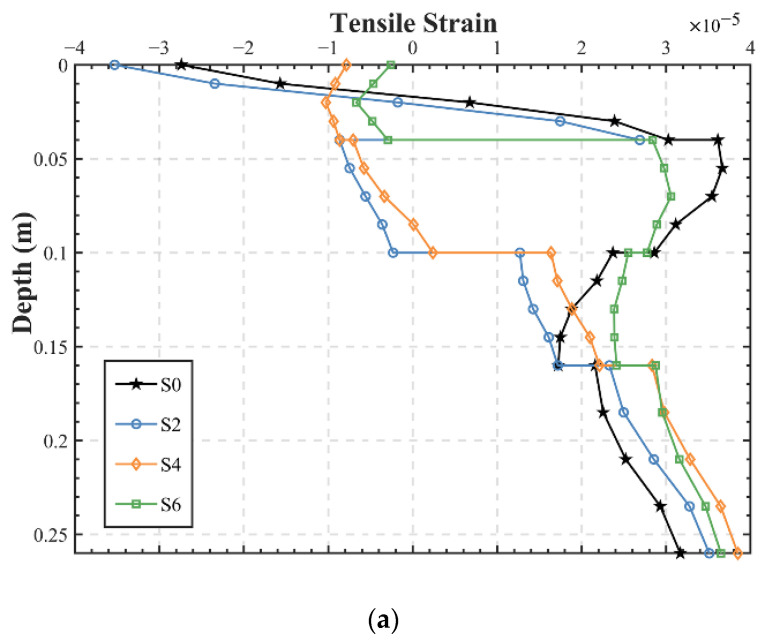

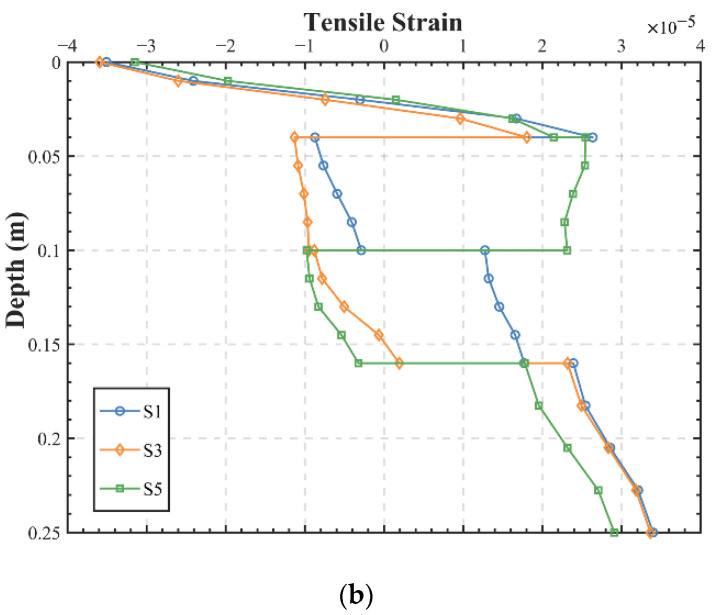
Pavement strain along depth: (**a**) S0, S2, S4, and S6; (**b**) S1, S3, and S5.

In addition to analysis of the maximum tensile strain at the bottom of the asphalt layer and tensile stress at the bottom of the base as design control indicators, the strain and stress characteristics of the whole asphalt pavement at loading peak are also of concern. As for strain characteristics, still using point A as the calculation point, the strain of the asphalt layer along depth was evaluated and classified with and without SAMI (Figure 7). It can be seen that the compressive strain in the upper 4 cm of S4 and S6 with EAC applied to the upper layer is significantly reduced and there is a more uniform strain distribution when compared to other structures. As the EAC layer transformed into an SUP layer, S4 and S6 underwent a sudden change in strain at 10 cm and 4 cm, respectively, from compressive strain to tensile strain. In the upper-middle layer (4–10 cm in depth), the same phenomenon occurs, where S0 and S6 without the application of EAC generate large tensile strains, while S2 and S4 still present a low compressive strain. Especially for S2, the original tensile strain in the upper layer increased sharply, while the strain in the upper-middle layer suddenly changed from tensile to compressive after the application of EAC. From this point of view, although S4 has a greater tensile strain at the bottom of the asphalt layer than the conventional structure, it might be argued that the EAC application solution for S4 is not unreasonable, because S4 is at a significantly lower strain level throughout the asphalt layer, which might be a considerable contribution to improving the overall fatigue life. Figure 7b shows a similar trend in that the tensile strain in the layer with EAC applied decreases remarkably, allowing for more uniform forces and deformations, which might lead to an alleviation of fatigue failure in the asphalt layer in some cases.

**Figure 8:**
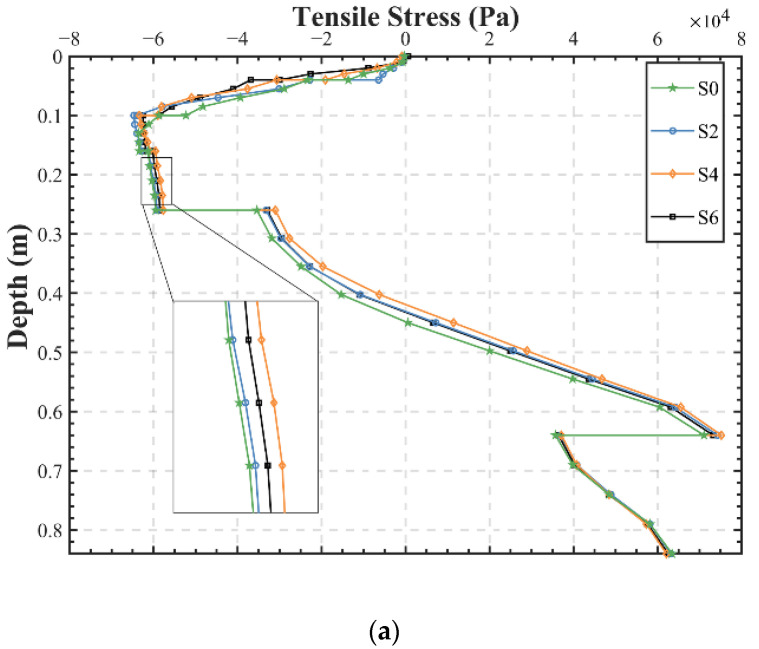

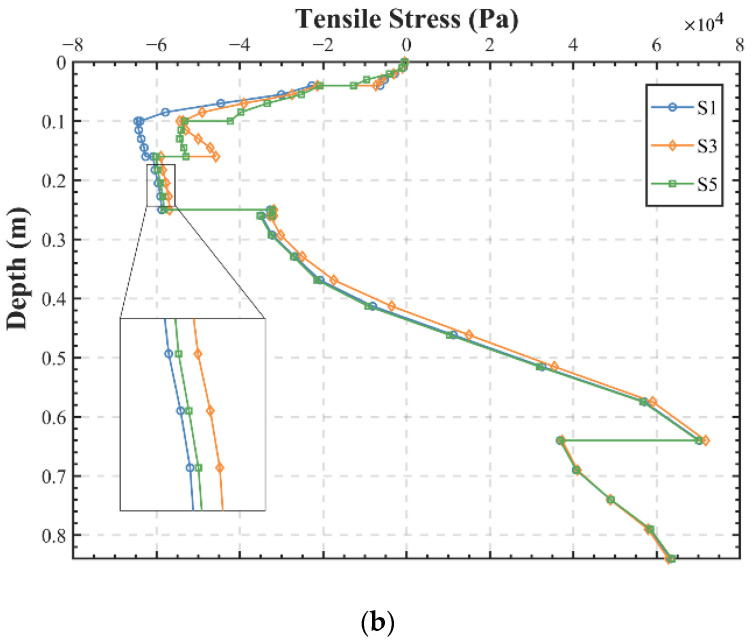
Pavement stress along depth: (**a**) S0, S2, S4, and S6; (**b**) S1, S3, and S5.

Figure 8 shows the stress distribution in the pavement structure, calculated using point C. It can be seen that different structures have little effect on the stress in the subgrade below the sub-base, and the impact is mainly distributed in the upper part of the pavement, especially in the asphalt layer. The trend of stress along depth is almost the same for all structures due to the fact that the changes in materials will significantly affect the stress–strain characteristics near its location (the asphalt layer), while having little impact on the overall structure at a distance. Meanwhile, the difference in the distribution of stress and strain in the asphalt layer might be caused by the variation of the calculation point locations. Compared to S0, the structures S2, S4, and S6 with EAC have less compressive stress in the asphalt layer, which is beneficial for the pavement. However, they have greater tensile stress in the lower part of the base, especially for S4, so when applying EAC, the issue of the fatigue life of the base should be considered and enhanced, and several methods such as strengthening the base materials and laying a bedding layer may be feasible.

Based on the previous calculations, more attention should be paid to structures with SAMI layers, as they may serve as a more reasonable solution. Figure 8b demonstrates that S5 not only has the lowest tensile strain at the bottom of the asphalt layer and tensile stress at the bottom of the base, but also has a lower value of tensile stress in the whole tensile region. In addition, its compressive stress is not excessively large, so S5 has a more balanced response and may possess the potential to be an engineering-suitable structure.

#### 3.2.4. Vertical Compressive Strain on the Top of Subgrade

**Figure 9:**
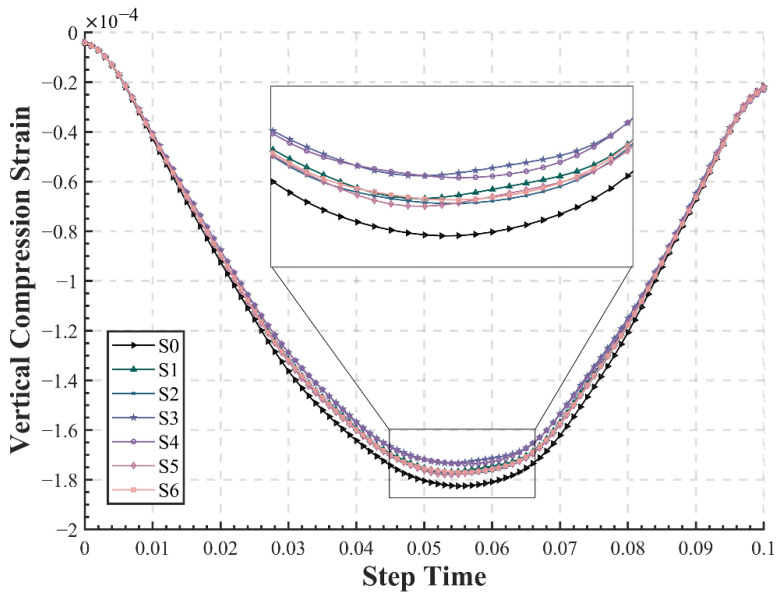
Time–history curve of vertical compressive strain on the top of subgrade.

The vertical compressive strain on the top of the subgrade plays an important role in the deformation of the soil and the generation of rutting. In this study, the point C on the top of the subgrade in the outermost lane was chosen as the calculation point for the vertical compressive strain (Figure 4d). For S0, S2, S4, and S6, it was Node 59 and Element 1027 and for S1, S3, and S5, it was Node 411 and Element 5111. The time–history curves and maximum values of vertical compressive strain on the top of the subgrade for different pavement structures are plotted and listed in Figure 9 and Table 6, respectively. The time–history curve is still identical to that of the loading pattern and is distributed as a sinusoidal curve. However, due to viscoelasticity, the vertical compressive strain also has a certain hysteresis effect, reaching its peak around 0.055 s, consistent with previous conclusions. Figure 9 illustrates that the vertical compressive strain presents three levels, with the conventional pavement S0 having the largest compressive strain, and the other two levels decrease by around 2.17% and 4.89%, respectively. S3 and S4 have the lowest value located in the first level, while S1, S2, and S5 are similar and lower than S0, lying in the middle level. It is interesting that all structures with EAC applied exhibited significantly smaller vertical compressive strains on the top of subgrade. This indicates that the application of EAC could substantially improve the rutting resistance of the pavement, which is in agreement with the findings of previous laboratory tests [44,45]. According to the laboratory results of wheel tracking tests, the dynamic stability of epoxy asphalt mixtures could be as high as 30,000 times/mm or more, presenting an extremely strong rutting resistance. This can also be verified by the three value levels in the figure. Compared with S1, S2, and S5, the use of double layers of EAC in S3 and S4 further reduces the vertical compressive strain on the top of subgrade, which further delays the generation of rutting on the pavement. This suggests that the proper use of EAC in pavement structures might be a way to reduce rutting distress, especially in high-temperature areas.

#### 3.2.5. Shear Stress in the Asphalt Layer

The shear stress in the asphalt layer has a non-negligible effect on the service life of asphalt pavements, although it is not currently used as a design indicator in China. At the same time, the shear stress in the upper and middle asphalt layers is also closely related to the appearance of rutting distresses, so the effect of shear stress in the asphalt layer should be investigated. The shear stress within the upper and upper-middle layers (0–10 cm) were mainly considered, and for the seven structures, the locations where the maximum values occur were selected as the calculation points.

**Figure 10:**
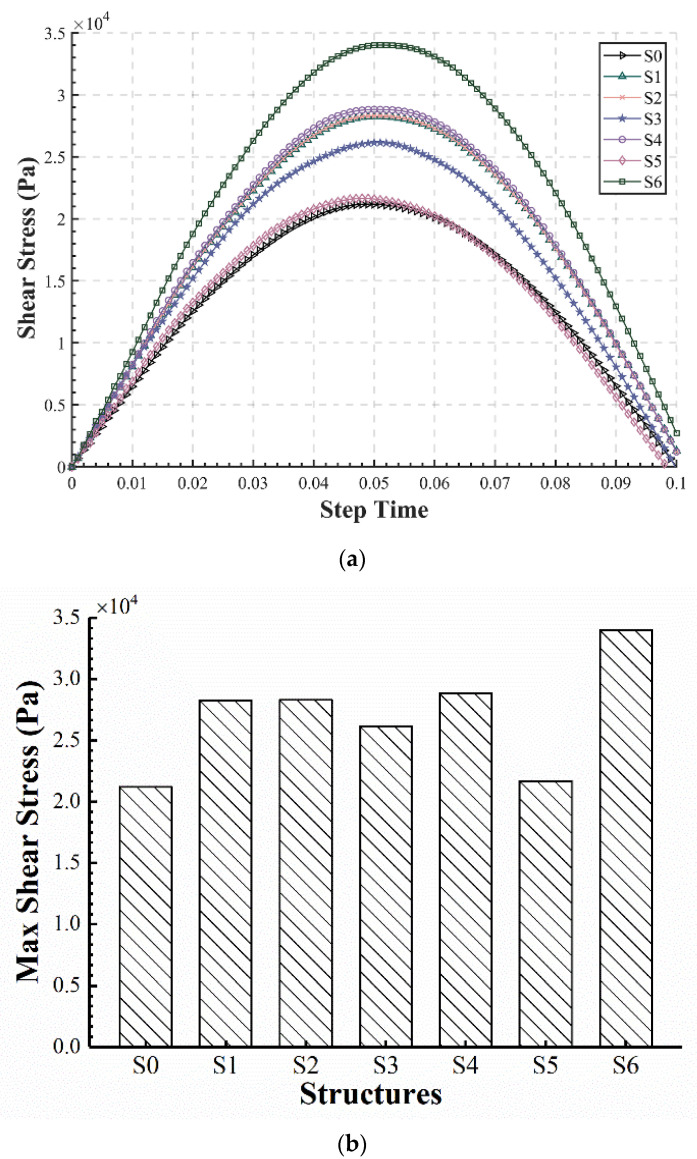
Shear stress in the asphalt layer: (**a**) Time–history curve; (**b**) maximum shear stress.

The time–history curves, maximum values and their locations of the shear stress were calculated, as shown in Figure 10 and Table 7. It can be seen that the shear stress varies greatly among different structures. S6 has a shear stress 1.6 times greater than the shear stress of S0, and S1, S2, and S4 have the same value of about 1.35 times the shear stress of S0. The increase in shear stress in S3 and S5, which have stress-absorbing layers with EAC in the lower-middle layers, is relatively less significant. The shear stress values of all structures with EAC applied are larger than S0, which may be due to the fact that the high stiffness of EAC changes the stress distribution, making the maximum value occur mostly in the layers where EAC is applied; this can also be verified from the vertical depths in Table 7. Therefore, even though EAC has the advantages of high strength and fatigue life, its shear resistance deserves heightened attention.

Additionally, the large value in S6 might be attributed to the application of EAC in the upper layer that is directly interacting with traffic loads, generating high local stress in the case of discontinuous pavement stiffness. A continuously applied EAC or an additional transition layer can be used to minimize this phenomenon. Among all the structures, the upside is that S5 has similar shear stress to S0, in spite of a slight increase. In S5, the EAC was applied to the lower layers near the base, so it has less of an effect on the stiffness continuity and the shear stress in the upper layers. From this perspective, it is more reasonable for EAC to be applied in the lower layer.

### 3.3. Prediction and Comparison of Design Life

**Figure 11:**
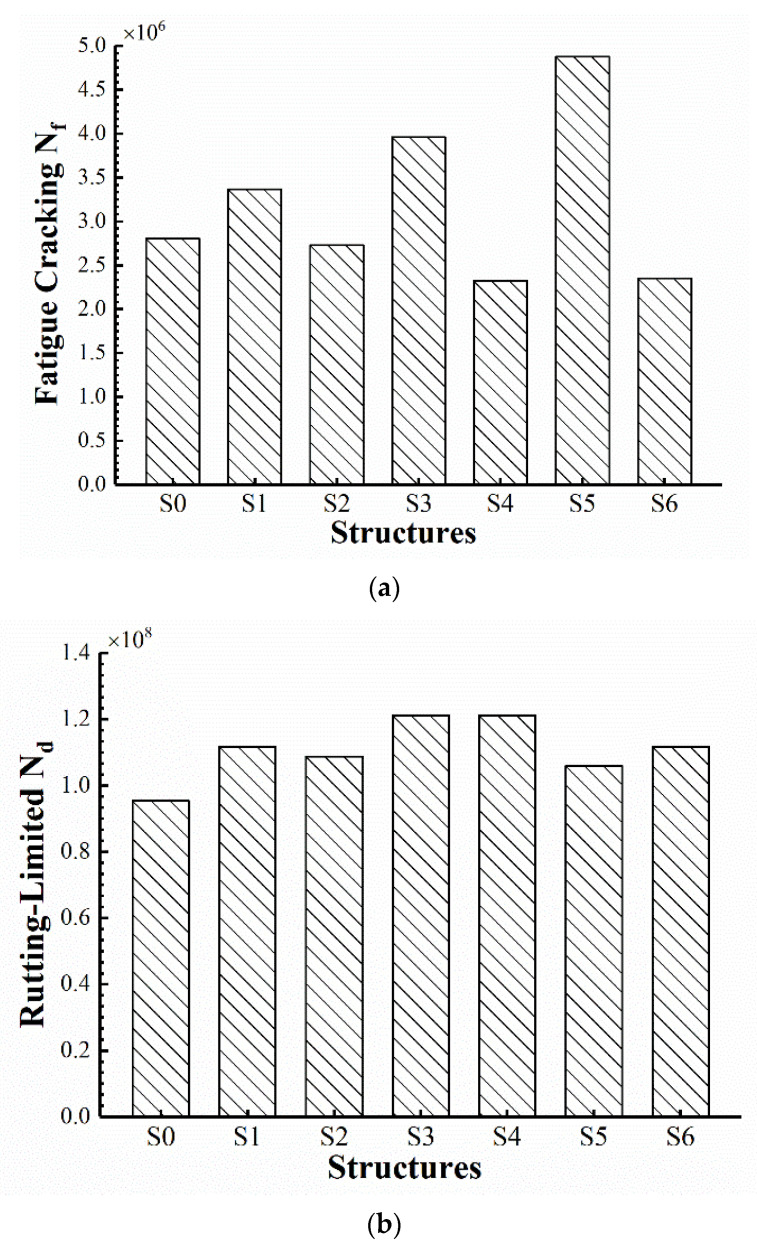
The allowable load repetitions: (**a**) $N_{f}$ to limit fatigue cracking; (**b**) $N_{d}$ to limit rutting.

Some researchers have used the empirical formulas from the America Asphalt Institute (AI) in previous studies [43], but only took into account two parameters, strain and modulus, leading to inaccuracy and large variability. In this paper, formulas in the Chinese specification JTG D50-2017 were used to calculate the design life, which incorporated more comprehensive parameters to achieve greater precision in the calculation. The allowable number of load repetitions to limit fatigue cracking and rutting of the seven structures is shown in Figure 11. With regard to the allowable number $N_{f}$ to limit fatigue cracking, S0 has a similar value to S2, while S4 and S6 are relatively lower. This indicates that EAC applied to the upper layer might affect the fatigue of the underlying asphalt mixtures, so the life of the overall structure needs to be taken into account, even though EAC has higher resistance. The structures with the SAMI layer (S1, S3, and S5) have significantly higher allowable load repetitions, especially S5, which allows for more than 1.7 times the fatigue number of conventional pavement S0, indicating that the epoxy asphalt pavement might have the potential to be a long-life pavement.

Meanwhile, the aging of the asphalt binder is also related to the expected service life of the asphalt mixture. In fact, the aging properties of the epoxy asphalt have been studied by several researchers, and it has been proven that the addition of epoxy components can provide epoxy asphalt with much better aging resistance. Apostolidis et al. [46] tracked the formation of carbonyl compounds by Fourier transform infrared (FTIR) spectroscopy and found that its reaction rate decreased when the epoxy components were added to the asphalt, which indicates that oxidative aging in epoxy asphalt occurs relatively slowly. Si et al. [47] proposed that aging changed the structure of the epoxy asphalt and improved its stress tolerance, increasing the tensile strength to 5.16 MPa. Additionally, after long-term aging, the fatigue life of EAC-13 is still three to four times that of AC-13, which is far superior to that of conventional asphalt concrete [22]. In terms of UV aging, the addition of the epoxy components gives the epoxy asphalt strong anti-aging properties, with a 12% increase in the residual penetration ratio [48]. From this point of view, the aging resistance of epoxy asphalt might be an advantage, or at least not much of a concern. As for the changes of material properties during use, a feasible approach in practical cases is to install sensors in the pavement and adjust the calculation of service life according to the real-time stress–strain data.

As for the allowable load repetitions $N_{d}$ to limit rutting, it can be seen that all structures with EAC (S0–S6) can withstand a higher number of loads than conventional pavement S0, indicating that they have much better rutting resistance, which is consistent with the results of laboratory wheel tracking tests on epoxy asphalt mixtures. Meanwhile, the load repetitions of rutting limits greatly exceed the fatigue cracking limits; therefore, the high-temperature performance of EAC might require less concern. However, despite the high resistance to fatigue cracking, S5 has a relatively weak rutting resistance, which could be further improved, though it still exceeds that of conventional pavement and the specification requirements.

### 3.4. Life Cycle Assessment (LCA) of Epoxy Asphalt Pavements

**Figure 12:**
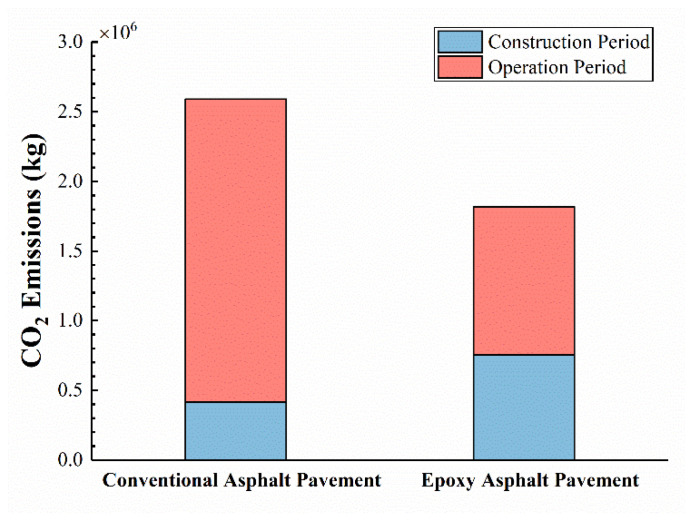
LCA of carbon emissions for epoxy asphalt pavement and conventional asphalt pavement.

The LCA results on carbon emissions of epoxy asphalt pavements and conventional asphalt pavements are shown in Figure 12. Due to the high production emissions of epoxy resin, the carbon emissions in the construction period of epoxy asphalt pavements are higher than those of conventional pavements but could already be significantly reduced by the lower mixing temperatures studied in this paper. More importantly, due to the longer service life and better performance, the epoxy asphalt pavements exhibit much lower carbon emissions from maintenance and repair during the operation period, about 48% of that of conventional pavements. As mentioned above, the extension of the service life of the pavement is a remarkably effective way to reduce emissions. It is critical to reduce pavement distresses and maintain good service condition, thereby significantly decreasing the carbon emissions generated by repairs. Thus, over the whole life cycle, epoxy asphalt pavements have an excellent emission reducing effect, reducing carbon emissions by 29.8% compared to conventional pavements. From the LCA results, the low-emission mixed EAC has strong feasibility as a long-life, “green”, and low-carbon pavement material.

## 4. Conclusions

In this study, the feasibility of low-emission mixed epoxy asphalt mixtures was verified, and six pavement structures with EAC were proposed for performance evaluation and compared to conventional pavements. Based on the viscoelastic parameters obtained from the dynamic modulus tests, an FEM model were developed to analyze the mechanical responses and the design life in order to conduct structure optimization. Finally, life cycle assessment on the carbon emissions of the recommended epoxy asphalt pavement structures was conducted. The following conclusions can be drawn:

1. Through the viscosity tests, the SBS-modified epoxy asphalt has a suitable mixing and compaction viscosity of 150 °C, so the mixing temperature of SBS-modified asphalt can be reduced by 30 °C after adding epoxy components. Thus, the SBS-modified epoxy asphalt can be considered to be a low-emission mixed material.
2. S5 with EAC applied in the middle-lower layers has the lowest tensile strain at the bottom of the asphalt layer and lowest tensile stress at the bottom of the base, decreasing by 11.81% and 1.54% compared to S0, respectively; however, S4 and S6 with EAC applied have an increasing trend in the upper layer.
3. The application of AR-SAMI as a stress-absorbing layer can significantly reduce the maximum tensile strain and stress in the asphalt layer, which might improve the fatigue life of the pavement. Additionally, the layer with EAC applied showed a remarkable decrease in tensile strain, resulting in more uniform stresses and deformations in the depth direction.
4. S3 and S4 reduced the vertical compressive strain on the top of the subgrade by 4.89%, indicating that the reasonable use of EAC could be a way reduce rutting distresses. The application of EAC increases the shear stress in the asphalt layer, especially when applied to the upper layer, while it has little increase in S5 compared to the conventional structure.
5. The allowable number of load repetitions to limit fatigue cracking of S5 is 1.7 times higher than that of the conventional structure, suggesting that it might have the potential to act as a long-life pavement. All structures with EAC have improved the allowable number to limit rutting, indicating that EAC has excellent rutting resistance.
6. The LCA results proved that in the 25 year life cycle, the carbon emissions of epoxy asphalt pavements could be reduced by 29.8% compared with the conventional pavements, showing a superior emission reduction effect.

In conclusion, the use of EAC, which allows for low-emission mixing, could enhance the performance and service life of the pavement, and has a favorable carbon emission reduction effect. Considering the performance and cost, structure S5 with EAC in the middle-lower layer is recommended. Future research can focus on simulations of fatigue cracking, top-down cracking, and rutting in different structures with EAC, while test sections in the field are also needed to obtain field stress–strain data to calibrate the accuracy of the simulation.

## Figures and Tables

**Table 1:** Properties of asphalt binder, epoxy resin, and curing agent [33].

Materials	Indicators	Values	Materials	Indicators	Values
Asphalt binder	Penetration (0.1 mm)	51.5	Epoxy resin	Density (g/cm^3^)	1.13
	Softening point (°C)	81.7		Viscosity (Pa•s, 25 °C)	4.1
	Ductility (cm, 5 °C)	29.2	Curing agent	Density (g/cm^3^)	0.92
	Kinematic Viscosity (Pa•s, 135 °C)	2		Viscosity (Pa•s, 25 °C)	0.2

**Table 2:** Gradation of EAC-13.

**Sieve size (mm)**	16	13.2	9.5	4.75	2.36	1.18	0.6	0.3	0.15	0.075
**Passing Percentage (%)**	100	95	76.5	53	37	26.5	19	13.5	10	6

**Table 3:** Prony series parameters of EAC and other asphalt mixtures.

EAC-13/16		SMA-13 *		SUP-20 **		SUP-25 **	
$\tau_{i}$	$G_{i}$ $\;\mathrm{or}\;K_{i}$	$\tau_{i}$	$G_{i}$ $\;\mathrm{or}\;K_{i}$	$\tau_{i}$	$G_{i}$ $\;\mathrm{or}\;K_{i}$	$\tau_{i}$	$G_{i}$ $\;\mathrm{or}\;K_{i}$
0.00001	0.02538	0.00001	0.17312	0.00001	0.16223	0.00001	0.16223
0.0001	0.01741	0.0001	0.21719	0.0001	0.20595	0.0001	0.20595
0.001	0.05832	0.001	0.20891	0.001	0.20636	0.001	0.20636
0.01	0.11526	0.01	0.14639	0.01	0.15432	0.01	0.15432
0.1	0.19804	0.1	0.07766	0.1	0.08746	0.1	0.08746
1	0.25276	1	0.03453	1	0.04104	1	0.04104
10	0.17329	10	0.01363	10	0.01682	10	0.01682
100	0.07532	100	0.00527	100	0.00671	100	0.00671
1000	0.03208	1000	0.00179	1000	0.00234	1000	0.00234
10,000	0.01114	10,000	0.00078	10,000	0.00092	10,000	0.00092
100,000	0.00575						

* SMA-13 = stone mastic asphalt with NMAS of 13 mm. ** SUP-20/25 = Superpave with NMAS of 20/25 mm.

**Table 4:** Summary of 7 pavement structures.

Structure Number	Pavement Structure
S0 (control group)	4 cm SMA-13 + 6 cm SUP-20 + 6 cm SUP-20 + 10 cm SUP-25
S1	4 cm SMA-13 + ***6 cm EAC-16*** + 6 cm SUP-20 + 9 cm SUP-25 + 1 cm AR-SAMI *
S2	4 cm SMA-13 + ***6 cm EAC-16*** + 6 cm SUP-20 + 10 cm SUP-25
S3	4 cm SMA-13 + ***6 cm EAC-16*** + ***6 cm EAC-16*** + 9 cm SUP-25 + 1 cm AR-SAMI
S4	***4 cm EAC-13*** + ***6 cm EAC-16*** + 6 cm SUP-20 + 10 cm SUP-25
S5	4 cm SMA-13 + 6 cm SUP-20 + ***6 cm EAC-16*** + 9 cm SUP-25 + 1 cm AR-SAMI
S6	***4 cm EAC-13*** + 6 cm SUP-20 + 6 cm SUP-20 + 10 cm SUP-25

* AR-SAMI = asphalt rubber stress-absorbing membrane interlayer, applied between SUP-25 and CTB.

**Table 5:** Material parameters of ABAQUS model.

Materials	Thickness (cm)	Modulus (MPa)	Poisson’s Ratio	Density (kg/m^3^)
EAC-13	4/6	2500	0.25	2400
EAC-16	4/6	2300	0.25	2400
SMA-13	4	1500	0.25	2400
SUP-20	6	1400	0.25	2400
SUP-25	9/10	1150	0.25	2400
AR-SAMI	1	200	0.3	1150
CTB	38	1500	0.25	2300
LFS	20	700	0.3	1800
SG	800	80	0.4	1800

**Table 6:** Summary of maximum indicators and their occurrence time.

Structure	S0	S1	S2	S3	S4	S5	S6
Max tensile strain at the bottom of asphalt layer	$5{.90\;\times \;10}^{-5}$	$5{.72\;\times \;10}^{-5}$	$6{.03\;\times \;10}^{-5}$	$5{.49\;\times \;10}^{-5}$	$6{.28\;\times \;10}^{-5}$	$5{.21\;\times \;10}^{-5}$	$6{.26\;\times \;10}^{-5}$
Occurrence time	0.060	0.062	0.063	0.062	0.063	0.062	0.064
Max tensile stress at the bottom of base (Pa)	71190	70330	74412	71828	75352	70095	73367
Occurrence time	0.052	0.051	0.051	0.051	0.05	0.051	0.051
Max vertical compressive strain on the top of subgrade	$1{.84\;\times \;10}^{-4}$	$1{.78\;\times \;10}^{-4}$	$1{.79\;\times \;10}^{-4}$	$1{.75\;\times \;10}^{-4}$	$1{.75\;\times \;10}^{-4}$	$1{.80\;\times \;10}^{-4}$	$1{.78\;\times \;10}^{-4}$
Occurrence time	0.055	0.053	0.055	0.053	0.056	0.053	0.055

**Table 7:** Maximum values and locations of shear stress in the asphalt layer.

Structure	S0	S1	S2	S3	S4	S5	S6
Maximum shear stress (Pa)	21,190.9	28,266.6	28,303.3	26,153.7	28,823.6	21,655.2	34,016.1
Element number	1918	5738	1891	5741	1715	5761	1714
Vertical depth (cm)	4	5.5	5.5	7	4	8.5	3
Horizontal points	Lane Center (Point C)	Center of the loading area (Point A)					

## Data Availability

The data presented in this study are available on request from the corresponding author.
